# Comparison of Gleason upgrading rates in transrectal ultrasound systematic random biopsies versus US-MRI fusion biopsies for prostate cancer

**DOI:** 10.1590/S1677-5538.IBJU.2017.0552

**Published:** 2018

**Authors:** Paulo Priante Kayano, Arie Carneiro, Tiago Mendonça Lopez Castilho, Arjun Sivaraman, Oliver Rojas Claros, Ronaldo Hueb Baroni, Rodrigo Gobbo Garcia, Guilherme Cayres Mariotti, Oren Smaletz, Renne Zon Filippi, Gustavo Caserta Lemos

**Affiliations:** 1 Hospital Israelita Albert Einstein, São Paulo, SP, Brasil; 2 Memorial Sloan Kettering Cancer Center - USA, New York, NY, EUA

**Keywords:** Prostatic Neoplasms, Magnetic Resonance Spectroscopy, Image-Guided Biopsy

## Abstract

**Purpose:**

Ultrasound-magnetic resonance imaging (US-MRI) fusion biopsy (FB) improves the detection of clinically significant prostate cancer (PCa).

We aimed to compare the Gleason upgrading (GU) rates and the concordance of the Gleason scores in the biopsy versus final pathology after surgery in patients who underwent transrectal ultrasound (TRUS) systematic random biopsies (SRB) versus US-MRI FB for PCa.

**Materials and Methods:**

A retrospective analysis of data that were collected prospectively from January 2011 to June 2016 from patients who underwent prostate biopsy and subsequent radical prostatectomy. The study cohort was divided into two groups: US-MRI FB (Group A) and TRUS SRB (Group B).

US-MRI FB was performed in patients with a previous MRI with a focal lesion with a Likert score ≥3; otherwise, a TRUS SRB was performed.

**Results:**

In total, 73 men underwent US-MRI FB, and 89 underwent TRUS SRB. The GU rate was higher in Group B (31.5% vs. 16.4%; p=0.027). According to the Gleason grade pattern, GU was higher in Group B than in Group A (40.4% vs. 23.3%; p=0.020). Analyses of the Gleason grading patterns showed that Gleason scores 3+4 presented less GU in Group A (24.1% vs. 52.6%; p=0.043).

The Bland-Altman plot analysis showed a higher bias in Group B than in Group A (-0.27 [-1.40 to 0.86] vs. −0.01 [-1.42 to 1.39]).

In the multivariable logistic regression analysis, the only independent predictor of GU was the use of TRUS SRB (2.64 [1.11 – 6.28]; p=0.024).

**Conclusions:**

US-MRI FB appears to be related to a decrease in GU rate and an increase in concordance between biopsy and final pathology compared to TRUS SRB, suggesting that performing US-MRI FB leads to greater accuracy of diagnosis and better treatment decisions.

## INTRODUCTION

Brazilian data shows that prostate cancer (PCa) is the most common non-cutaneous malignancy in men. The estimated incidence of PCa for 2016 is 61,200 new cases, with an estimated risk of 61.82 new cases per 100,000 men (1). PCa is commonly diagnosed by transrectal ultrasound (TRUS)-guided random biopsies. The Gleason score of PCa has been shown to be an important criterion to predict tumour behaviour and to determine the appropriate course of treatment (2). However, the randomness and non-targeted nature of TRUS biopsy can result in inaccurate sampling of the cancer and misclassification of cancer risk. Researchers have demonstrated discrepancies in the Gleason score of TRUS biopsy compared to the final surgical specimens, with under-estimation reported in approximately 30% of cases (3).

Recently, multiparametric magnetic resonance imaging (mpMRI) of the prostate has been shown to be valuable in the detection, localization and characterization of prostatic tumour foci (4). Target biopsy of the abnormality detected by MRI was initially performed by cognitive guidance of the topographic location of the cancer, but in recent years, devices were developed to combine ultrasound and mpMRI images. The US-MRI fusion images can guide biopsy and improve the detection of clinically significant prostate cancer (5, 6). A more accurate diagnostic method is desirable to avoid misclassification, which is particularly important in appropriate decision-making for the treatment of PCa (active surveillance or focal therapy or radical treatment). It is plausible that the Gleason score misclassification and upgrading noted in radical prostatectomy specimens can be reduced by employing more accurate biopsy techniques.

The aim of this study was to compare the Gleason upgrading rates and the concordance of the biopsy versus final pathology Gleason scores in patients who underwent TRUS systematic random biopsies (SRB) versus US-MRI fusion biopsies (FB) for prostate cancer.

## MATERIALS AND METHODS

### Patient selection and data collection

We included all consecutive patients who underwent prostate biopsy and subsequent radical prostatectomy at our institution. The study cohort was divided into those who had US-MRI FB (from June 2013 to July 2015) (Group A) and TRUS SRB (from June 2010 to February 2015) (Group B). Pathological analyses of the prostatectomy specimens were reviewed by a single, experienced pathologist and were considered as the standard of reference. Patients who did not undergo biopsy and radical prostatectomy at our institution and those with pathologic specimens that were not reviewed by the same pathologist were excluded to avoid bias. IRB approval and a waiver for informed consent were obtained for this retrospective study using prospectively collected data from our institution database.

US-MRI FB was performed in all patients with mpMRI-detected abnormalities and Likert scores ≥3. Patients with a normal mpMRI or Likert scores <3 underwent TRUS SRB. All radical prostatectomies were performed by our institution Urology staff either by robot-assisted radical prostatectomy or by an open approach.

Data related to clinical, biopsy, histopathological and MRI characteristics were collected.

### Multiparametric MRI

MRIs were performed on 3T scanners (Siemens Prisma 3T, Siemens PetRM 3T, GE 750W 3,0T, Philips 3,0T) with a phased-array coil and included high-resolution T2-weighted imaging, diffusion-weighted imaging and dynamic contrast-enhanced imaging. A Likert scale score, that is a subjective assessment on the likelihood of the presence of prostate cancer on a 5 point scale (7), was assigned by one of our uro-radiologist with years of experience in interpreting prostate MRI (median of 7 years of experience; range 5 to 15 years) and every exam were reviewed by other experienced radiologist, and if there was a discrepancy in the analyses, the score was assigned after a consensus. Only lesions classified with scores ≥3 were defined as targets for US-MRI fusion biopsy.

### US-MRI Fusion Biopsy

Targeted biopsies were performed by our institution interventional radiologist team, with experience in non-elastic fusion prostate biopsy and experience in reading prostate MRI (median of 9 years of experience with TRUS SRB and 2 years with target US-MRI FB) using different US-MRI fusion systems: MyLab 60 (Esaote, Florencea, Italy), Aplio 500 Smartfusion (Toshiba, Nasu, Japan) and Logiq E9 VNav (GE Healthcare, Milwaukee).

Each biopsy was performed with the patient in a left lateral decubitus position, using endocavitary 4 to 9 MHz broadband curved array end-fire transducers and an 18-gauge side-notch cutting core biopsy needle (20-mm stroke length). Patients first underwent systematic 14- core biopsies (six from each lobe and one more from each transitional zone), followed by targeted biopsies generally consisting of 2 or 3 cores from each target.

### Histopathology

Gleason scoring was performed according to the 2005 International Society of Urological Pathology consensus recommendations (8). We classified the patients according to the 2014 ISUP consensus meeting held in Chicago in 2014, which classify the Gleason scores into grade groups (Gleason score ≤6 = ISUP 1; Gleason score 3+4 = ISUP 2; Gleason score 4+3 = ISUP 3; Gleason score 4+4 = ISUP 4; Gleason score 9 or 10 = ISUP 5) (9).

Cores from each lesion were numbered and labelled according to the target, enabling radiology-pathology correlation in patients with multiple targets.

Surgical specimens were processed using a modified Stanford technique; 3- to 5-mm transverse sectioned samples were taken from the apex to the base and from the sagittal section of the distal 5 to 8 mm of the apex and base.

### Statistical analysis

The primary endpoint of this study was to compare the rate of any Gleason score upgrade of RP compared to US-MRI fusion biopsy and random biopsy alone. Descriptive statistics were used for patient characteristics. An independent Student's *t*, Mann-Whitney, chi-square or Fisher's exact test was used to compare characteristics of the patients when appropriate. Gleason upgrading was compared by comparison of proportions. A multivariable logistic regression using forced entry was carried out to assess the independent predictors of Gleason upgrading. The results are presented as odds ratios (ORs) and 95% confidence intervals. Agreement between Fusion US-MRI and histopathology and Random Biopsy and histopathology was assessed using a Bland-Altman plot, and bias was calculated with their respective 95% confidence intervals.

All analyses were conducted with SPSS v.20 (IBM SPSS Statistics for Windows, Version 20.0. Armonk, NY: IBM Corp.) or R v.2.12.0 (R Foundation for Statistical Computing, Vienna, Austria). For all analyses, two-sided *p* < 0.05 were considered significant.

## RESULTS

### Characteristics of the cohort

A total of 73 men who underwent US-MRI fusion biopsies and 89 who underwent TRUS systematic random biopsies were included in our analyses. In both groups, the patient demographics were similar (Table-1). There were no differences according to prostate volume (histopathology), PSA, clinical and pathologic staging and number of lymph nodes. However, there was a significant difference in biopsy Gleason score between both groups. Patients from Group A had fewer Gleason score 6 tumours (11% vs. 28%), and patients from Group B had greater total tumour volumes (15% vs. 10%) and fewer clinically significant tumours (70.8% vs. 89%) (Table-1), defined as patients with Gleason score greater or equal to 3+ 4 or greater than ISUP 1.

**Table 1:** Patients characteristics.

		US-MRI fusion	Random	*p* value
		(n = 73)	(n = 89)	
Age (years), median (IQR)		65.0 (57.5 – 69.0)	64 (59 – 69)	0.838
PSA (ng/mL), median (IQR)		4.8 (3.7 – 6.4)	5.5 (4.2 – 7.2)	0.060
Prior biopsy status, n (%)		9 / 73 (12.3)	10 / 89 (11.2)	0.829
Biopsy Gleason grade group, n (%)				
	Less or equal to 6	8 / 73 (11.0)	25 / 89 (28.1)	
	3+4 = 7	29 / 73 (39.7)	19 / 89 (21.3)	0.007
	4+3 = 7	21 / 73 (28.8)	23 / 89 (25.8)	
	8	8 / 73 (11.0)	18 / 89 (20.2)	
	9-10	7 / 73 (9.6)	4 / 89 (4.5)	
Number of total cores, median (IQR)		18.0 (12.0 – 19.5)	15.0 (14.0 – 17.5)	0.144
Number of random cores, median (IQR)		14.0 (11.0 – 18.0)	14.0 (14.0 – 17.0)	0.171
Number of targeted cores, median (IQR)		4.0 (3.0 – 5.0)	ND	
Positive cores, n (%)		61 / 71 (85.9)	13 / 20 (65)	0.007
Positive targeted cores, median (IQR)		6.0 (4.5 – 10.0)	ND	
Prostate volume at histopathology (grams), median (IQR)		42.0 (30.0 – 56.0)	40.0 (32.5 – 47.0)	0.223
Surgical specimen Gleason grade group, n (%)				
	Less or equal to 6	2 / 73 (2.7)	9 / 89 (10.1)	
	3+4 = 7	32 / 73 (43.8)	29 / 89 (32.6)	0.205
	4+3 = 7	28 / 73 (38.4)	31 / 89 (34.8)	
	8	5 / 73 (6.8)	8 / 89 (9.0)	
	9-10	6 / 73 (8.2)	12 / 89 (13.5)	
Total tumor volume (%), median (IQR)		10.0 (7.0 – 20.0)	15.0 (10.0 – 20.0)	0.024
Bilateral tumor, n (%)		54 / 73 (74.0)	67 / 89 (75.3)	0.848
Multifocal tumor, n (%)		60 / 73 (82.2)	76 / 89 (85.4)	0.580
Positive lymph node, n (%)		1 / 70 (1.4)	3 / 87 (3.4)	0.424
Clinically significant tumor, n (%)		65 / 73 (89.0)	63 / 89 (70.8)	0.004
Time between biopsy and surgery (days), median (IQR)		30.0 (30.0 – 60.0)	60.0 (30.0 – 60.0)	0.224
Numbers of lesions on MRI, median (IQR)		1.0 (1.0 – 2.0)	ND	---

### Primary endpoint

Gleason upgrading was higher in patients who underwent TRUS SRB compared to US-MRI FB (31.5% vs. 16.4%; *p*=0.027) (Table-2). When analysing Gleason upgrading according to the Gleason grade group, there was also a higher rate of Gleason upgrading in patients who underwent TRUS SRB compared to those who underwent US- -MRI FB (40.4% vs. 23.3%; *p*=0.020) (Table-2). Analyses from separate Gleason grade groups showed that Gleason scores of 3+4 presented less Gleason upgrading in the FB group (24.1% vs. 52.6%; *p*=0.043) (Table-2). Finally, there was no difference in Gleason upgrading when considering only patients with Gleason scores of 6 on biopsy (Table-2).

**Table 2:** Gleason upgrading.

		Fusion (n = 73)	Random (n = 89)	*p* value
Biopsy Gleason score		7.0 (7.0 – 7.0)	7.0 (6.0 – 7.5)	0.137
Surgical specimen Gleason score		7.0 (7.0 – 7.0)	7.0 (7.0 – 7.0)	0.765
Gleason upgrading*		12 / 73 (16.4)	28 / 89 (31.5)	0.027
Gleason upgrading in patients with Gleason 6 on biopsy**		6 / 8 (75.0)	17 / 26 (65.4)	0.611
Gleason upgrading according to Gleason grade pattern		17 / 73 (23.3)	36 / 89 (40.4)	0.020
	3+3	6 / 8 (75.0)	16 / 25 (64.0)	0.687
	3+4	7 / 29 (24.1)	10 / 19 (52.6)	0.043
	4+3	3 / 21 (14.3)	3 / 23 (13.0)	1.000
Accuracy***		51 / 73 (69.9)	56 / 89 (62.9)	0.353

* defined as the number of patients with Gleason score in surgical specimen greater then biopsy sample.

** defined as the number of patients with Gleason score 6 in biopsy sample (clinically non-significant disease) that presented a Gleason score greater than 6 in surgical specimen (clinically significant disease). Patients with Gleason score greater than 6 were excluded from this analyses.

*** defined as the number of patients who presented the same Gleason score in biopsy sample and surgical specimen.

**Figure 1:**
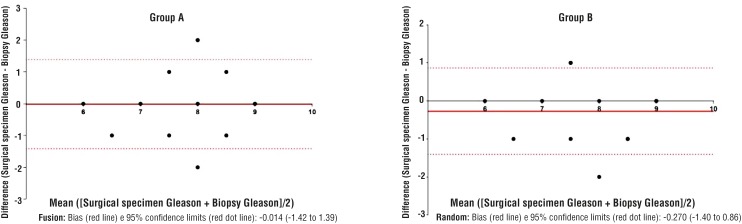
Bland - Altman Plot Group A vs. Group B.

The Bland-Altman plot analysis showed a higher bias for patients submitted to TRUS systematic random biopsy compared to those submitted to US-MRI fusion biopsy (-0.27 [-1.40 to 0.86] vs. −0.01 [-1.42 to 1.39]) (Figure-1). In the multivariate logistic regression, the use of TRUS systematic random biopsy, compared to US-MRI fusion biopsy, was the only independent predictor of Gleason upgrading (2.64 [1.11 – 6.28]; p=0.024) (Table-3).

**Table 3:** Logistic Regression (outcome: Gleason upgrading).

	Univariate		Multivariate	
	OR (95% CI)	p value	OR (95% CI)	*p* value
Age	1.01 (0.97 – 1.06)	0.596	---	---
Baseline PSA	1.05 (0.94 – 1.19)	0.371	1.03 (0.91 – 1.17)	0.632
Prostate volume	0.99 (0.97 – 1.01)	0.277	0.99 (0.96 – 1.01)	0.282
Time between biopsy and surgery	1.00 (0.99 – 1.01)	0.358	0.99 (0.97 – 1.01)	0.173
LIKERT score	1.26 (0.56 – 2.81)	0.575	---	---
Random biopsy	2.33 (1.09 – 5.01)	0.030	2.64 (1.11 – 6.28)	0.028

**PSA =** prostate-specific antigen; **OR =** odds ratio; **CI =** confidence interval

The comparison analysed by the Bland- -Altman plot of Group A and Group B showed that the agreement bias between Gleason score on biopsy and Gleason score on surgical specimen was lower in Group A (Figure-1).

## DISCUSSION

In our study, we found a significantly lower rate of Gleason upgrading using US-MRI FB, showing that this method can improve prostate cancer characterization at biopsy. All mpMRI, biopsies and surgical specimens were evaluated by the same team and methods to keep the pattern and to reduce bias in the Gleason and Likert score classifications.

The use of US-MRI FB was associated with a lower rate of Gleason upgrading compared to the use of TRUS SRB (16.4% vs. 31.5%; *p*=0.027). In the clinical setting, a diagnostic tool that can determine the “true” Gleason score plays a crucial role in guiding the clinician in making the best therapeutic decision, mainly for low-and intermediate-risk PCa. In the active surveillance scenario, it is imperative to decrease the risk of missing a high-grade disease and delaying a radical treatment, providing more confidence to the urologist and patient with conservative management of PCa.

For patients who will be treated by prostatectomy or radiation therapy, the biopsy Gleason score is considered a key point in most nomograms for determining the indication of extended lymphadenectomy and changes in the irradiation field or the time of hormone therapy in patients under radiation therapy, thus changing the impact on the morbidity (10-14).

Data reported in the literature show that the Gleason score is frequently lower for TRUS guided biopsies compared to that for surgical specimens, with under-estimation reported in about 30% of cases. In patients with low-grade prostate biopsies, the risk of upgrading may increase up to 50% (3).

Our study showed that patients with initial Gleason scores of 6 presented more Gleason upgrading in the US-MRI FB group (75% vs. 65.4%; *p*=0.611); however, this difference was not statistically significant, probably due to the relatively small number of patients. Biopsies with Gleason scores of 3+4 presented less Gleason upgrading in the US-MRI FB group (24.1% vs. 52.6%; *p*=0.043). The concordance was higher when the highest Gleason grading pattern was analysed between biopsy and surgical specimens, consistent with our hypothesis that US- -MRI FB increases the detection of the highest- -grade tumour (3, 15, 16).

Arsov et al. reported an MRI FB and TRUS random biopsy upgrading of 21.2% and 32.7%, respectively (17). In this study, Gleason upgrading was twice as frequent in patients who underwent US-MRI FB compared with TRUS SRB (31.5% versus 16.4%).

Multiparametric MRI of the prostate has shown its value in the detection, localization and characterization of prostatic tumour foci (4) and plays an important role to avoid unnecessary biopsies in patients with previously negative ones, showing accuracies of approximately 90% for the diagnosis of significant prostate cancer (18-20).

In our institution, most urologists are using mpMRI in biopsy-naive patients, avoiding some biopsies in patients with low probability of clinically significant prostate cancer, which can explain the lower rate of Gleason 6 in patients who underwent US-MRI FB (11% vs. 28.1%)

Prostate mpMRI and target biopsy could be incorporated into active surveillance selection criteria, having a higher accuracy for risk stratification (21). Prostate mpMRI can also reduce the need for repetitive biopsies by as much as 68% through non-invasive serial monitoring for those on active surveillance (22). Disease reclassification on those in active surveillance with normal mpMRI appears to be very low, with negative predictive value ranges from 81-90% (23-25).

One limitation of this study is the non- -randomized retrospective study design and the heterogeneous population studied, which might lessen the generalizability of our results because of potential selection bias. Another possible selection bias is the small number of patients with Gleason 6 biopsy scores, because we perform mpMRI in biopsy naive patients and avoid the biopsy on those with LIKERT 1 or 2. A prospective randomized study might eliminate this bias and might confirm our hypothesis.

## CONCLUSIONS

US-MRI FB appears to be associated with a lower Gleason upgrading rate and a higher concordance between biopsy and final pathology compared to TRUS SRB, leading to greater accuracy of diagnosis and therefore better treatment decisions. The routine use of MRI before biopsy is associated with a decrease in the detection of clinically insignificant tumours.
